# An integrated analysis of QTL mapping and RNA sequencing provides further insights and promising candidates for pod number variation in rapeseed (*Brassica napus* L.)

**DOI:** 10.1186/s12864-016-3402-y

**Published:** 2017-01-11

**Authors:** Jiang Ye, Yuhua Yang, Bo Chen, Jiaqin Shi, Meizhong Luo, Jiepeng Zhan, Xinfa Wang, Guihua Liu, Hanzhong Wang

**Affiliations:** 1 Oil Crops Research Institute of the Chinese Academy of Agricultural Sciences, Key Laboratory of Biology and Genetic Improvement of Oil Crops, Ministry of Agriculture, Wuhan, 430062 China; 2 College of Life Science and Technology, Huazhong Agricultural University, Wuhan, 430070 China

**Keywords:** *Brassica napus*, Pod number, QTL mapping, RNA sequencing, DEG, Candidate gene

## Abstract

**Background:**

As the most important yield component in rapeseed (*Brassica napu*s L.), pod number is determined by a series of successive growth and development processes. Pod number shows extensive variation in rapeseed natural germplasm, which is valuable for genetic improvement. However, the genetic and especially the molecular mechanism for this kind of variation are poorly understood. In this study, we conducted QTL mapping and RNA sequencing, respectively, using the BnaZNRIL population and its two parental cultivars Zhongshuang11 and No.73290 which showed significant difference in pod number, primarily due to the difference in floral organ number.

**Result:**

A total of eight QTLs for pod number were identified using BnaZNRIL population with a high-density SNP linkage map, each was distributed on seven linkage groups and explained 5.8–11.9% of phenotypic variance. Then, they were integrated with those previously detected in BnaZNF_2_ population (deriving from same parents) and resulted in 15 consensus-QTLs. Of which, seven QTLs were identical to other studies, whereas the other eight should be novel.

RNA sequencing of the shoot apical meristem (SAM) at the formation stage of floral bud primordia identified 9135 genes that were differentially expressed between the two parents. Gene ontology (GO) analysis showed that the top two enriched groups were S-assimilation, providing an essential nutrient for the synthesis of diverse metabolites, and polyamine metabolism, serving as second messengers that play an essential role in flowering genes initiation. KEGG analysis showed that the top three overrepresented pathways were carbohydrate (707 genes), amino acid (390 genes) and lipid metabolisms (322 genes).

*In silico* mapping showed that 647 DEGs were located within the confidence intervals of 15 consensus QTLs. Based on annotations of *Arabidopsis* homologs corresponding to DEGs, nine genes related to meristem growth and development were considered as promising candidates for six QTLs.

**Conclusion:**

In this study, we discovered the first repeatable major QTL for pod number in rapeseed. In addition, RNA sequencing was performed for SAM in rapeseed, which provides new insights into the determination of floral organ number. Furthermore, the integration of DEGs and QTLs identified promising candidates for further gene cloning and mechanism study.

**Electronic supplementary material:**

The online version of this article (doi:10.1186/s12864-016-3402-y) contains supplementary material, which is available to authorized users.

## Background

Pod number is one of the three yield components (pod number per plant, seed number per pod and seed weight) and breeding targets in rapeseed (*Brassica napu*s L.). Among the three components of yield in rapeseed, pod number shows the highest correlation with yield, which suggests it to be the major contributor to yield [1]. Pod number, especially from branch inflorescence and whole plant, is also the most variable among the three yield components, in accordance with its modest heritability observed in most studies [2, 3]. In rapeseed germplasm resources, pod number shows extensive natural variation, which is invaluable for the genetic improvement [4]. However, the genetic and especially the molecular mechanism for this kind of variation are poorly understood.

Pod number is also a very complex trait that is multiplicatively determined by its three components: the number of flowers differentiated, the proportion of ovaries successfully fertilized, and the rate of fertilized ovaries developed into pods, which are determined by the flower bud differentiation, fertilization and pod development, respectively. Of these, flower bud differentiation is the first critical developmental stage and morphogenesis process which determines the number of floral organs [5]. This process is undertaken with the interaction of internal (such as carbohydrates, phytohormones, polyamine etc.) and external (such as light, temperature, water, and fertilizer etc.) factors [5]. Whereas, genetic factors are the most important control combined with internal signals (also genetically controlled) and influenced by environmental interactions. During the reproductive phase, the shoot apical meristem (SAM) produces inflorescence meristem (IM) that quickly develops into floral meristems (FMs) that, in turn, produce floral primordia [6]. In *Arabidopsis*, more than 100 regulators had been characterized to involve in SAM growth and development [6–10]. In addition, a complex regulatory network of hormones, transcription factors, enzymes, microRNA and other cellular components had been developed [11]. In rice, several genes involving in the regulation of floral organ number had been isolated, such as *FON1*-*FON4* [12–15]. However, molecular mechanism of floral bud differentiation remained relatively unclear in *Brassica napus*.

Pod number is a typical quantitative trait, which shows continuous variation and is very sensitive to the environmental conditions [16]. To the present, more than 80 pod number quantitative trait loci (QTLs) have been identified from nearly ten linkage mapping populations [4]. However, only a few of these QTLs were repeatedly detected, in accordance with the modest heritability of pod number. In addition, almost all of these pod number QTLs showed a moderate effect [4]. Therefore, it is difficult to narrow down these pod number QTLs and identify the underlying candidate genes.

The previous expression profiling studies largely relied on hybridization-based technologies, which was unable to fully catalogue and quantify the diverse RNA molecules that are expressed from genomes over a wide range of levels [17]. With the rapid advancement and decreased price of high-throughput sequencing technology in recent years, RNA sequencing has been widely applied in various species to conduct annotation and quantification of all genes and their isoforms across samples [18]. However, a large amount of differentially expressed genes (DEGs) were usually identified between contrast samples through RNA sequencing. Therefore, integration of DEGs and QTLs is considered to be a promising method to identify potential candidate genes [19].

In our previous study, we screened two rapeseed cultivars Zhongshuang11 and No.73290 that showed significant difference in pod number [4] mainly due to the difference in floral organ number. The main objectives of our study are to: (1) dissect the genetic mechanism of pod number variation by QTL mapping using BnaZNRIL and BnaZNF_2_ population derived from Zhongshuang11 and No.73290; (2) dissect the molecular mechanism of pod number variation by characterizing the expression profile and DEGs of SAM for Zhongshuang11 and No.73290 using RNA sequencing technology; (3) integrate the QTLs and DEGs to identify potential candidate genes for further functional and mechanism studies.

## Results

### Mapping of QTLs for pod number using BnaZNRIL population

**Fig. 1:**
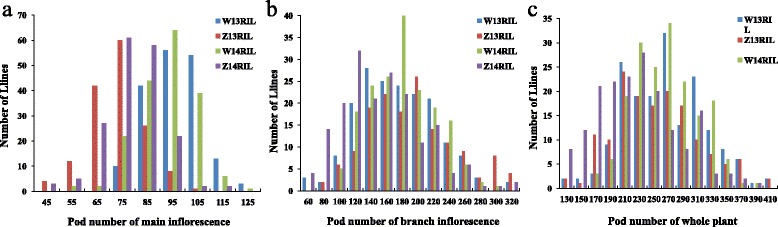
Distribution of pod number in the BnaZNRIL population in the four environments. The horizontal axis represented the value of pod number for the main inflorescence (**a**), branch inflorescence (**b**), and whole plant (**c**). The vertical axis represented the number of lines within the BnaZNRIL population. The different experiments were represented by different colors as showed in the legend. W13RIL, Z13RIL, W14RIL and Z14RIL were the codes of the four experiments

The pod number of No.73290 was much larger than that of Zhongshuang11 in all of the three investigated environments (Table 1). The pod number of the BnaZNRIL population showed normal or near-normal distribution in the four environments (Fig. 1), indicating a quantitative inheritance suitable for QTL mapping [4]. The broad-sense heritability was 0.67, 0.62 and 0.62 for pod number of main inflorescence, branch inflorescence and whole plant, respectively (Additional file 1: Table S1).

**Table 1:** Pod number for the two parents and derived RIL populations in the four investigated environments

Environment	Materials		PNm	PNb	PNw
W13	Parents	Zhongshuang11	71	130	201
		No.73290	107	211	306
		Pt-test	3.82E-05	4.98E-03	4.41E-04
	RIL	Min	62	39	115
		Max	136	356	458
		Mean	92	165	257
Z13	Parents	Zhongshuang11	60	104	163
		No.73290	87	230	322
		Pt-test	5.56E-05	9.83E-03	2.96E-03
	RIL	Min	22	58	88
		Max	99	480	566
		Mean	68	183	246
W14	Parents	Zhongshuang11	78	101	179
		No.73290	108	146	237
		Pt-test	5.42E-07	3.96E-03	4.88E-03
	RIL	Min	45	45	126
		Max	121	362	419
		Mean	87	170	255
Z14	Parents	Zhongshuang11	66	102	178
		No.73290	/	/	/
		Pt-test	/	/	/
	RIL	Min	33	28	75
		Max	129	390	491
		Mean	74	143	216
Total	Parents	Zhongshuang11	71	110	184
		No.73290	100	192	280
		Pt-test	1.38E-12	2.72E-07	3.81E-08
	Population	Min	22	28	75
		Max	136	480	566
		Mean	81	164	243

PNm/PNb/PNw was the abbreviation of pod number from the main inflorescence, branch inflorescence and whole plant. W13, Z13, W14 and Z14 were the codes of the four environments

**Fig. 2:**
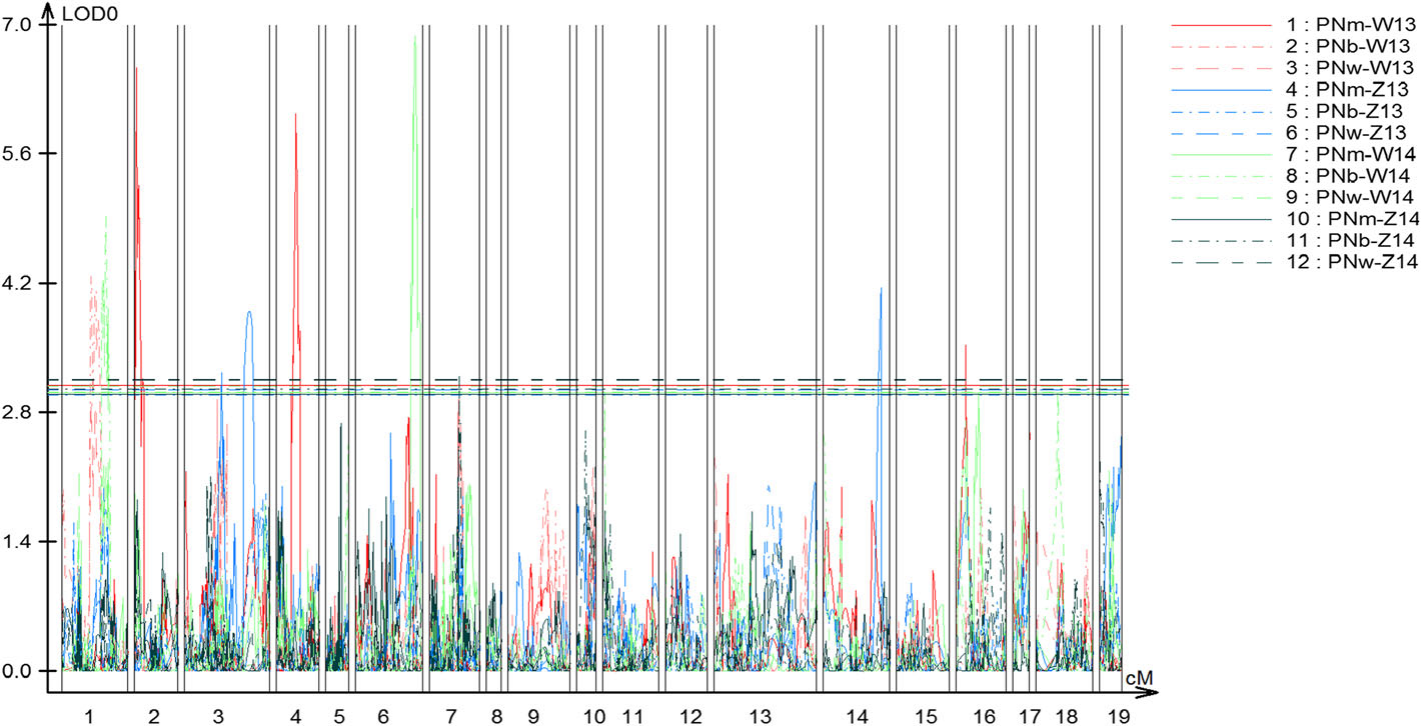
QTL scanning curves for pod number in the BnaZNRIL population. The horizontal axis represented the no. of the 19 linkage groups (A1-A10; C1-C9). The vertical axis represented the LOD score. PNm/PNb/PNw was the abbreviation of pod number from the main inflorescence, branch inflorescence and whole plant. W13, Z13, W14 and Z14 were the codes of the four experiments. The straight lines on the middle of the figure indicated the LOD threshold values corresponding to *P* = 0.1

A high-density linkage map comprising 2264 SNP markers was constructed using the BnaZNRIL population. After deleting two non-reproducible suggestive QTL, a total of eight QTLs were identified for pod number in four experiments, six and two for main inflorescence (PNm) and branch inflorescence (PNb), respectively (Fig. 2). The identified QTLs were distributed on A01 (*qPN.A01-2, −3,*), A02 (*qPN.A02-1*), A03 (*qPN.A03-3*), A04 (*qPN.A04-1*), A06 (c*qPN.A06-1*), C04 (*qPN.C04-1*) and C06 (*qPN.C06-2*) linkage groups. These QTLs for PNb and PNm explained 9.3–9.9% and 5.8–11.9% of phenotypic variance, respectively. The additive effect of these QTLs for PNb and PNm ranged from −16.3 to −13.8 and from −4.1 to 2.5, respectively. Furthermore, the corresponding genomic intervals of the eight QTLs were determined through mapping the probe sequences of SNP markers within their confidence intervals to the *Brassica napus* genome (Additional file 2: Table S2).

Because QTL mapping of pod number had been previously performed using the BnaZNF_2_ population derived from the same parents [4], meta-analysis was carried out to integrate pod number QTLs detected in the two studies. After meta-analysis, a total of 15 consensus-QTLs were obtained (Table 2), of which seven were identical to the previously identified ones, whereas the other eight should be novel (Additional file 3: Table S3).

**Table 2:** The 15 consensus- QTLs of pod number obtained after meta-analysis from BnaZNF_2_ and BnaZNRIL population

QTL	Peak position	Confidence interval	Genomic region(Mb)	Additive effect	LOD value	*R* ^2^ (%)	Experiments code
*qPN.A01-2*	74.9	61.9–85.8	4.4–8.7	−16.3	4.3	9.3	W13RILb
*qPN.A01-3*	98.4	87.2–103.9	8.7–15.7	−13.8	4.9	9.9	W14RILb
*qPN.A01-1*	104.4	94.3–105.8	15.3–18.3	−4.9	3.0	12.5	W10F_2:3_m/X11F_2:3_m
*qPN.A02-1*	4.2	1.9–5.3	0.1–0.4	−4.1	6.5	11.2	W13RILm
*qPN.A03-1*	61.5	57.5–61.9	11.4–13.5	−9.7	3.6	3.0	W11F_2:3_w
*qPN.A03-2*	81.6	64.6–98.0	14.2–15.4	−1.7	3.8	3.4	W11F_2:3_m
*qPN.A03-3*	142.7	130.5–155.5	15.5–20.8	−3.6	3.9	10.5	Z13RILm
*qPN.A04-1*	41.9	37.0–51.3	11.5–13.3	−3.3	6.0	10.2	W13RILm
*qPN.A05-1*	10.8	4.0–13.8	6.0–8.0	−4.7	3.4	12.6	W09F_2_m
*cqPN.A06-1*	/	/	22.0–23.7	−5.6	7.9	20.6	W10F_2:3_bmw/W11F_2:3_bmw/X11F_2:3_bmw/X11F_2:4_bmw/W14RILm
*qPN.A09-1*	44.0	42.5–54.4	17.6–24.0	4.1	2.7	2.3	X11F_2:3_bw
*qPN.C02-1*	8.2	3.2–21.5	1.3–2.5	−5.1	4.3	18.5	W10F_2:3_m/W11F_2:3_m/X11F_2:3_m
*qPN.C04-1*	128.5	119.2–129.6	36.7–45.7	−3.3	4.2	8.7	Z13RILm
*qPN.C06-1*	51.2	49.1–53.0	17.9–18.8	7.3	4.4	15.0	W10F_2:3_m/W11F_2:3_m
*qPN.C06-2*	20.2	20.1–20.3	29.3–31.3	2.5	3.5	5.8	W13RILm

W13, Z13, W14 and Z14 were the codes of the four environments

Because the flowering time of the two parents (Zhongshuang11 and No.73290) for both BnaZNF_2_ and BnaZNRIL populations differed for about one week, therefore the 15 pod number consensus-QTLs were also compared with the flowering time QTLs detected in both populations. The results showed that most of the QTLs for both traits were not overlapped (Shi et al. unpublished data).

Based on rapeseed genome annotation [20], a total of 6164 genes resided in regions of 15 QTLs. Number of rapeseed genes in QTL region ranged from 63 (*qPN.A02-1*) to 1103 (*qPN.C04-1*), with the mean of 411 (Table 3). The majority of these genes have never been functionally annotated in *Brassica napus*.

**Table 3:** Number of genes, DEGs and meristem-related genes within the QTL region

QTL	Gene	DEG	
		Total	Related to meristem development
*qPN.A01-2*	781	74	0
*qPN.A01-3*	675	74	1
*qPN.A01-1*	338	43	0
*qPN.A02-1*	63	11	0
*qPN.A03-1*	375	40	1
*qPN.A03-2*	288	33	2
*qPN.A03-3*	939	51	0
*qPN.A04-1*	280	34	0
*qPN.A05-1*	248	23	1
*cqPN.A06-1*	289	43	0
*qPN.A09-1*	223	33	0
*qPN.C02-1*	212	62	2
*qPN.C04-1*	1103	69	0
*qPN.C06-1*	82	15	0
*qPN.C06-2*	268	42	2

Gene represented total rapeseed genes. DEGs represented the differently expressed genes between Zhongshuang11 and No.73290. Homologous represented meristem-related genes

### Transcriptome sequencing and mapping

To dissect the possible molecular mechanism of pod number difference between Zhongshuang11 and No.73290, transcriptome sequencing was performed using the shoot apical meristem (SAM) of Zhongshuang11 and No.73290 because their pod number difference was mainly attributable to the floral organ number. Total RNA, isolated from SAM of ten individuals of Zhongshuang11 and No.73290 at the formation stage of floral bud primordia, was pooled and transcribed into cDNA. Then, the two cDNA libraries were constructed and sequenced via Illumina Hiseq 2000 platform. Consequently, 172,006,032 and 170,055,032 raw reads were generated for Zhongshuang11 and No.73290, respectively. After removal of sequences with adaptor, duplication and low quality, we obtained 157,471,012 and 155,457,898 high-quality clean reads for Zhongshuang11 and No.73290, respectively. A summary of the transcriptome sequencing is exhibited in Table 4.

**Table 4:** Summary of the transcriptome sequencing

Sample name	Raw reads	Clean reads	Cleans bases	Error rate (%)	Q20 (%)	Q30 (%)	GC content (%)
Zhongshuang11	172,006,032	157,471,012	15,408,439,159	0.03	99.45	95.42	46.03
No.73290	170,055,032	155,457,898	15,207,889,681	0.03	99.45	95.39	46.13

A total of 127,275,824 (80.82%), and 125,875,780 (80.97%) clean reads for Zhongshuang11 and No.73290, respectively, were successfully mapped to the released reference genome [20] of *Brassica napus* using SOAPaligner/soap2 [21] (Table 5). Of which, 106,578,237 (67.68%) and 105,439,605 (67.83%) clean reads for Zhongshuang11 and No.73290, respectively, were uniquely mapped to the reference genome.

**Table 5:** The number and proportion of (uniquely) mapped reads among clean reads

Sample name	Mapped reads (Mapped/Clean)	Uniquely mapped reads (Unique/Clean)
Zhongshuang11	127,275,824 (80.82%)	106,578,237 (67.68%)
No.73290	125,875,780 (80.97%)	105,439,605 (67.83%)

### Identification and validation of DEGs between Zhongshuang11 and No.73290

**Fig. 3:**
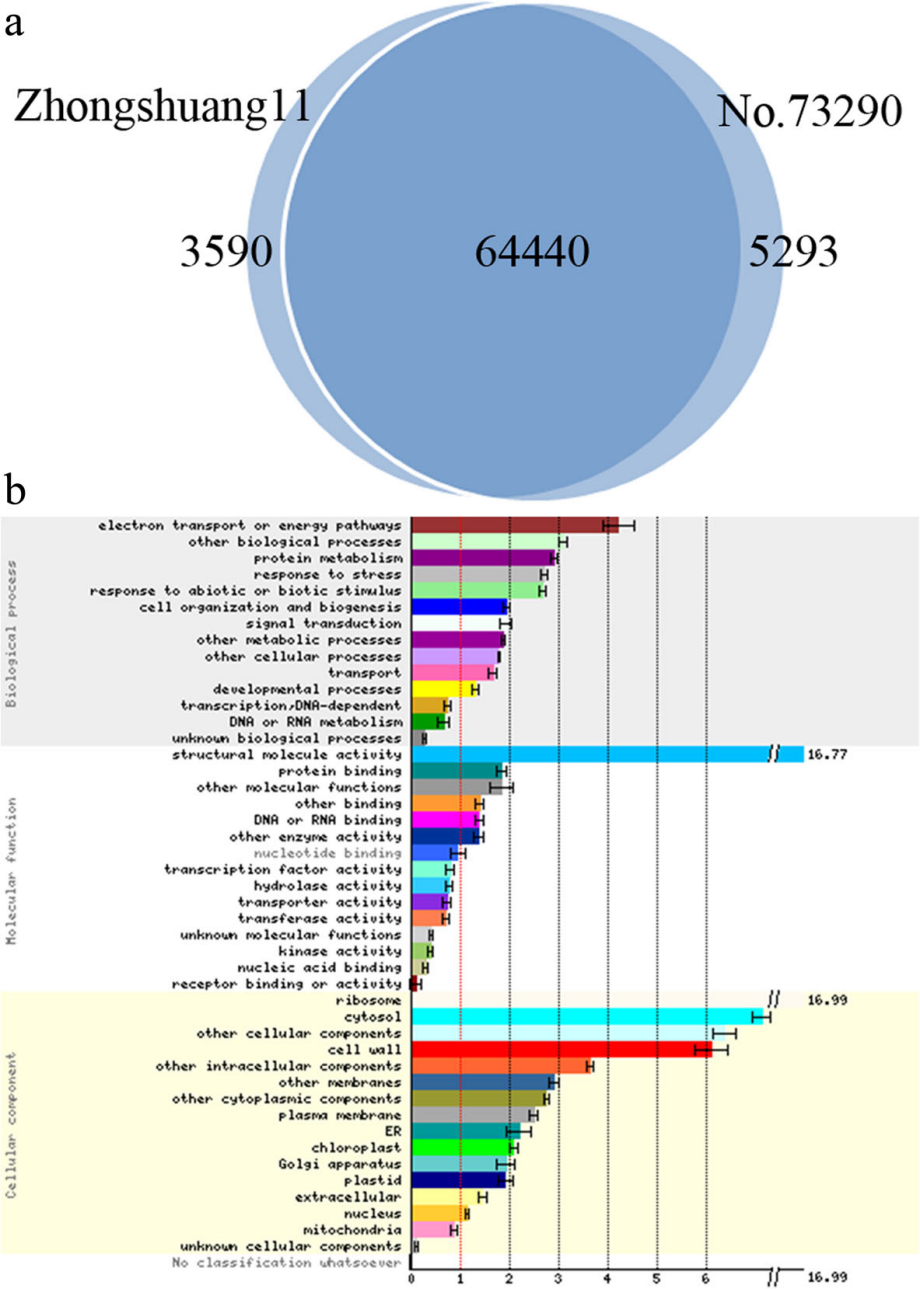
Characteristics of expression genes in SAM of Zhongshuang11 and No.73290. **a** The Venn diagram showing the overlaps of expression genes between Zhongshuang11 and No.73290. **b** GO analysis of expression genes with average RPKMs beyond 60 using the Classification SuperViewer Tool (http://bar.utoronto.ca/ntools/cgi-bin/ntools_classification_superviewer.cgi). Groups with gray words indicated unenriched GO terms (*p* ≥ 0.05)

All the uniquely mapped reads were selected for further analysis. To remove technical biases inherent in the sequencing process, most notably the length of the RNA species and the sequencing depth of samples, RPKM (reads per kilobase per million reads) was used to normalize these measures [22]. A total of 68030 (67.33%) and 69733 (69.02%) genes expression were detected for Zhongshuang11 and No.73290 respectively. Notably, 64440 (87.89%) genes expression was commonly detected in both Zhongshuang11 and No.73290 (Fig. 3a). Genes with average RPKMs beyond 60 were selected to conduct gene ontology (GO) analysis (Fig. 3b). These genes involved in biological processes including electron transport or energy pathway, protein metabolism and other biological process. The most enriched category in molecular function was structural molecule activity. The most overrepresented category in cellular component were ribosome, followed by cytosol, other cellular components and cell wall.

Using false discovery rate (FDR) < 0.001 and fold change > 2 as the significant level, a total of 9135 differentially expressed genes (DEGs) were identified. Of which, 5507 (60.28%) and 3628 (39.72%) DEGs were respectively up and down regulated, in No.73290 compared to Zhongshuang11 (Additional file 4: Table S4).

**Fig. 4:**
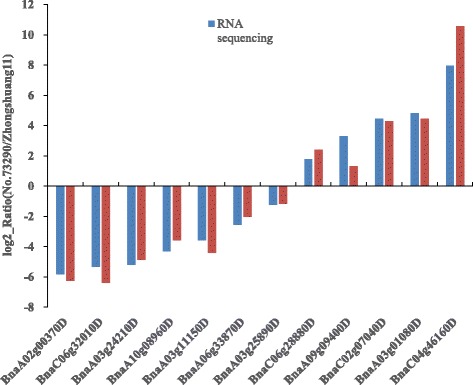
Comparison of the relative expression abundance measured by qRT-PCR and RNA-seq for 12 selected DEGs

To validate the results of RNA sequencing, qRT-PCR was conducted on 12 randomly selected DEGs with 7 down-regulated and 5 up-regulated genes. As shown in Fig. 4, the expression patterns for 12 DEGs were generally consistent between RNA sequencing and qRT-PCR data. Whereas, differences in fold changes measured by RNA sequencing and qRT-PCR were observed, which is consistent with other studies [23].

### Functional annotation of the DEGs

All the DEGs were annotated based on their similarity (E value ≤1e-5) in six public databases, including GO, KEGG, Pfam, InterPro, SwissProt, and TrEMBLE (Table 6). In total, 8479 DEGs (92.82%) have significant similarity in at least one database.

**Table 6:** Summary of functional annotation for total DEGs in six public databases

	No. of DEGs hits	Percentage (%)
Annotated in GO	5433	59.47
Annotated in KEGG	4576	50.09
Annotated in Pfam	7300	79.91
Annotated in InterPro	7082	77.53
Annotated in Swiss-Prot	6307	69.04
Annotated in Tremble	8433	92.32
Annotated in at least one database	8479	92.82
Total DEGs	9135	100

**Fig. 5:**
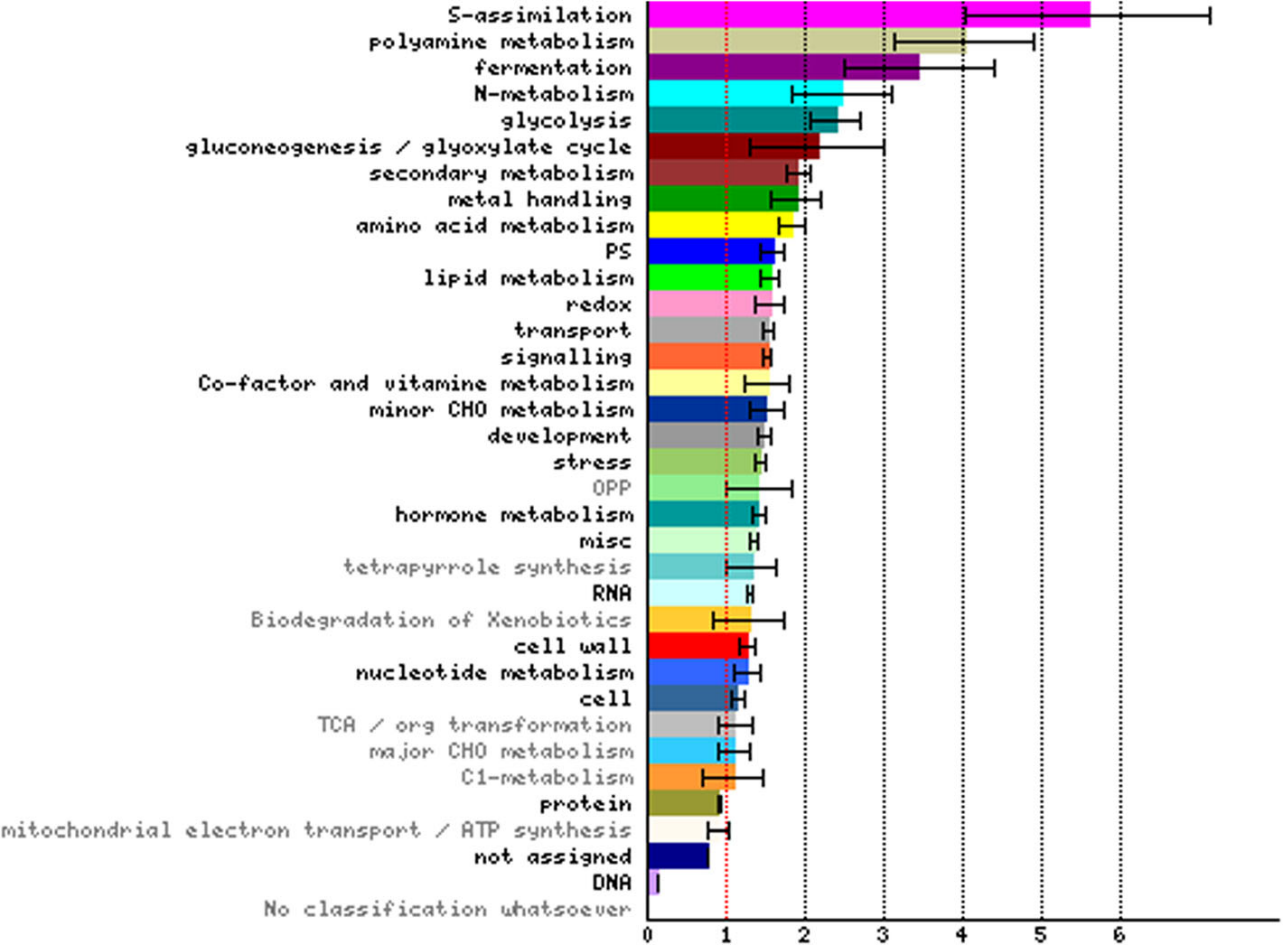
Gene functional classification of differentially expressed genes using the Classification SuperViewer Tool (http://bar.utoronto.ca/ntools/cgi-bin/ntools_classification_superviewer.cgi). The X axis shows the normalized class score (± bootstrap StdDev). Groups with gray words indicated unenriched GO terms (*p* ≥ 0.05)

Because the majority of the rapeseed reference genes have no functional annotation. These DEGs were then annotated by sequence alignment with those in TAIR 10 (E-value cutoff of 1e-5). Finally, 8305 (90.91%) of 9135 DEGs matched at least one gene in *Arabidopsis* (Additional file 5: Table S5). To overview functions of DEGs, the 8305 annotated DEGs were assigned to at least one Gene Ontology (GO) category that belonged to three major terms: cellular component, molecular function and biological process. After the absolute gene numbers in each group were normalized to the frequency of group over all *Arabidopsis* genes (Fig. 5), S-assimilation was the most over-represented group, indicating that the role of S-assimilation in SAM growth and development. In plant, the uptake of sulfate and its assimilation provides an essential nutrient for the synthesis of diverse metabolites, including cystesine, methionine, glutathione, vitamin cofactors and so on [24]. These metabolites affect carbon nitrogen ratio, which likely contributes to floral bud development [25]. The following over-represented group was polyamine metabolism. Previous studies indicated that polyamine served as second messengers playing an essential role in flowering genes initiation [26]. Other significantly enriched groups, such as N-metabolism, amino acid metabolism, lipid metabolism or hormone metabolism, displayed the active metabolic status of SAM.

**Fig. 6:**
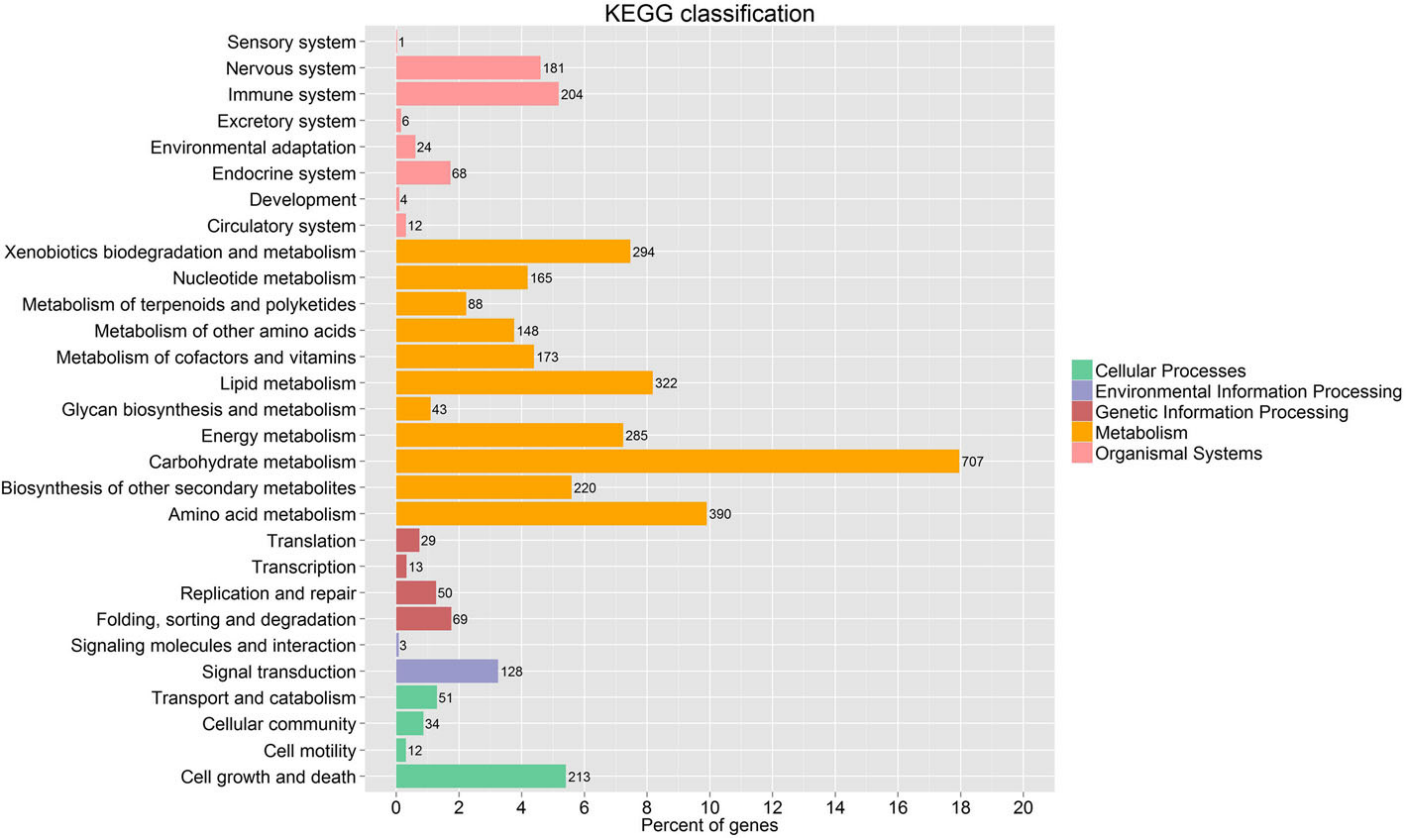
KEGG classification of the DEGs. Total pathways were grouped into five categories: cellular process, environmental information process, genetic information process, metabolism and organismal systems. Figures indicated the number of DEGs

To further study the biological pathways that related to SAM growth and development, the pathway enrichment analysis was conducted in the *Kyoto Encyclopedia of Genes and Genomes* (KEGG) pathway database. These DEGs were mapped to 190 KEGG pathways (Additional file 6: Table S6), which were grouped into five categories: cellular processes, genetic information processing, environmental information processing, metabolism, and organismal systems (Fig. 6). Of which, metabolism (2835 genes, 72.01%) was most enriched, which indicated that developmental differences of SAM between Zhongshuang11 and No.73290 were largely related to metabolism. Moreover, the top three represented pathways were carbohydrate metabolism (707 genes), amino acid metabolism (390 genes) and lipid metabolism (322 genes), which all belonged to the metabolism group. This is understandable, because the flower bud differentiation is dependent first on the nutrient level in the body of plant, which is reflected by the cytosol concentration (in the shoot apex growing point) that is determined by the metabolic process. Carbohydrate (as the structure and energy matter) accumulation is closely related to flower bud differentiation [5]. Increasing amino acid content promotes flower bud formation [27]. Lipid metabolism contributes to cell membrane and other parts. Because the SAM is a reservoir of undifferentiated stem cells that function as a continuous source of new cells [11]. These results provide further insight into the molecular mechanism responsible for floral organ number variation in rapeseed.

### Integration of DEGs with QTLs

To further understand the roles of these DEGs played in regulating SAM development and the final flower/pod number, they were integrated with the total of 15 pod number consensus-QTLs identified in both BnaZNRIL and BnaZNF_2_ population by *in silico* mapping. A total of 647 DEGs were located in the regions of the 15 QTLs, and the DEGs number within each QTL ranged from 11 (*qPN.A02-1*) to 74 (*qPN.A01-2/ qPN.A01-3*), with a mean of 43 (Table 3). Of which, 604 DEGs (93.35%) showed significant homology with *Arabidopsis* genes and the most have functional annotation.

Interestingly, nine DEGs underlying six QTLs were known to play an important role in regulating meristem growth and development. The nine DEGs resided in the regions of *qPN.A01-3(BnaA01g22100D), qPN.A03-1(BnaA03g25890D), qPN.A03-2(BnaA03g29180D, BnaA03g29810D), qPN.A05-1(BnaA05g12220D), qPN.C02-1(BnaC02g02900D, BnaC02g03640D),* and *qPN.C06-2(BnaC06g28880D, BnaC06g29980D)* (Table 7). Their functions included transcription factor, auxin receptor, enzyme, and other cellular elements. For example, *BnaA03g25890D* (underlying *qPN.A03-1*) is homologous to *Arabidopsis ABP1* (*AT4G02980*), which is an extra-cellar auxin receptor that involves in the maintenance of anisotropic growth to initiate new floral primordial [28]. *BnaC06g29980D* (underlying *qPN.C06-2*) is homologous to *Arabidopsis AP1* (*AT1G69120*), a floral homeotic gene encoding MADS-domain transcription factor, which plays an essential role in floral meristem determinacy, maintenance of floral meristem identity, flower development, and the transcriptional activation of several flowering time genes including *AG*, *SVP*, *SOC1* and *AGL24* [29, 30]. *BnaC02g02900D* (underlying *qPN.C02-1*) is homologous to *Arabidopsis TFL1* (*AT5G03840*), which is involved in floral initiation process and control floral meristem identity [31]. The nine genes should be considered as promising candidates that could be used for further study. Our study also indicated that integration of transcriptome sequencing with QTL mapping could be an efficient method to narrow down the number of candidate genes within the QTL region.

**Table 7:** Details of nine DEGs those are related to meristem development and within the QTL regions

QTL	Rapeseed gene	*Arabidopsis* gene	Gene function
*qPN.A01-3*	*BnaA01g22100D*	*AT1G60340*	Transcriptional regulator
*qPN.A03-1*	*BnaA03g25890D*	*AT4G02980*	Auxin binding protein
*qPN.A03-2*	*BnaA03g29180D*	*AT3G05840*	Kinase
	*BnaA03g29810D*	*AT3G07050*	Inflorescence meristem maintenance
*qPN.A05-1*	*BnaA05g12220D*	*AT2G30130*	Repress KNOX gene expression
*qPN.C02-1*	*BnaC02g02900D*	*AT5G03840*	Inflorescence meristem identity
	*BnaC02g03640D*	*AT5G02030*	Transcription factor
*qPN.C06-2*	*BnaC06g28880D*	*AT1G67770*	Mei2-like proteins
	*BnaC06g29980D*	*AT1G69120*	Transcription factor

## Discussion

### Novel QTLs identified for pod number

In the present study, a total of eight QTLs for pod number were identified in the BnaZNRIL population, and were integrated with those previously detected in BnaZNF_2_ population [4], which resulted in a total of 15 consensus-QTLs. These QTLs were distributed on ten linkage groups (A01, A02, A03, A04, A05, A06, A09, C02, C04 and C06). Most of these QTLs showed a moderate effect (*R* ^2^ < 10%) and only one (*cqPN.A06-1*) can be considered as a “major” QTL. Of these, only four QTLs were repeatedly detected and the other 11 were specific (Table 2). This strongly indicated that pod number is very sensitive to environmental condition, and accorded well with the moderate heritability of pod number found in the current and previous studies [2, 3].

More than 70 QTLs for pod number had been detected in the other studies, which were distributed on 17 (excluding C04 and C07) of the 19 linkage groups [4]. To accurately identify the positional relationship of pod number QTLs detected in our and other studies, comparative QTL analysis was performed based on the physical map of *Brassica napus* [4]. Of the total of 89 pod number QTLs identified in our and other studies, 83 ones were successfully mapped on the physical map. This represents the first relatively comprehensive genetic architecture of pod number in rapeseed. Of the 15 consensus-QTLs detected in our studies, the genomic regions of seven QTLs (*qPN.A01-2, qPN.A02-1, qPN.A03-1, qPN.A03-2, qPN.A03-3, qPN.C06-1 and qPN.C06-2*) were overlapped with those reported in other studies, whereas the other eight (*qPN.A01-3, cqPN.A01-1, qPN.A04-1, qPN.A05-1, cqPN.A06-1, qPN.A09-1, qPN.C02-1* and *qPN.C04-1*) should be novel. More importantly, we detected a pod number QTL (*qPN.C04-1*) on C04 linkage group for the first time.

### Characteristics of SAM DEGs

Transcriptome sequencing of SAM had been documented in several crops such as maize [32] and soybean [33], but had rarely been reported in *Brassica napus*. To our knowledge, this is the second report on SAM transcriptome sequencing in rapeseed. In this study, KEGG analysis showed that the most enriched group of these DEGs belong to metabolism, especially carbohydrate metabolism, which provides the energy and material basis to produce the difference of the number of differentiated flower buds and opening flowers. We proposed that these carbohydrates should be mostly transferred from the leaf, because it is the major source of photosynthesis during the vegetative and early reproductive stage.

To date, a total of 2296 transcription factors have been identified and classified into 58 families in *Arabido*psis (http://planttfdb.cbi.pku.edu.cn/index.php?sp=Ath). Based on the functional annotation of corresponding *Arabidopsis* homologous of these DEGs, nearly all transcription factors families (52 of the total 58 families) were found. This indicated that transcription factors may play an important role in SAM development. In addition, phytohormones metabolism was overrepresented. The roles of several types of hormones in SAM development have been extensively studied. Of which, cytokinins and auxins act as two major hormones to involve in meristem function and maintenance [34]. Auxins are a positive regulator to induce lateral organ initiation, such as flower bud, leaf. Cytokinins play a role in meristem maintenance and in controlling meristematic properties, such as cell proliferation. Furthermore, auxins interact with cytokinins to regulate SAM development [34, 35]. In the SAM, gibberellin activity is down-regulated and its activity is antagonistic with cytokinins activity [35]. In the SAM, brassinosteroids play a role in the spatiotemporal control of organ boundary formation and morphogenesis [36]. What’s more, phytohormones and transcription factors can cooperate to balance meristem maintenance and organ production [35]. These results showed a complex regulatory network involved in SAM development.

### Candidates for pod number

Previous studies into pod number mainly focused on QTL mapping (in genomics level) and no study has been conducted in transcriptomics. RNA sequencing is a new high-throughput, high-sensitivity and high-speed technology, which is widely used for transcriptome profiling [37]. In comparison with the previous methods, RNA sequencing possesses three key advantages: first, RNA sequencing is not limited to detect transcripts that correspond to existing genomic sequences; Second, RNA sequencing has very low background signal; Third, transcripts detected by RNA sequencing have a large dynamic range of expression levels [38]. The integration of the transcriptome profiling and QTL mapping is a very useful and popular strategy toward discovery of candidate genes [19].

Our previous study showed that pod number of No.73290 was about half greater than that of Zhongshuang11 [4], which was mainly due to their difference in floral organ number according to results of successive observation in several years. According to the relevant research, floral organ number was mainly determined by the SAM development [12, 39]. Thus, SAM of both Zhongshuang11 and No.73290 was characterized using the RNA sequencing technology and the identified DEGs was combined with QTLs to narrow the candidates.

According to the functional annotation of the corresponding *Arabidopsis* homologues of these DEGs, nine involved in meristem growth and development were considered to be the candidates, which should be important targets for further functional validation.

Transcription factors (such as *WUS, STM*) play a central role in SAM growth and development [11, 40]. Of the above-mentioned nine candidates, the *Arabidopsis* homologues (*AP1*, *BLH9*, and *AT1G60340*) of *BnaC06g29980D*, *BnaC02g03640D* and *BnaA01g22100D* were transcription factor-encoding genes. *AP1*, a MAD box transcription factor, activates floral organ identity genes to promote FMs formation by interplaying with *LFY* [29, 41, 42]. Loss function of *AP1* prevents the formation of flowers in the axils of sepal to form FMs [43]. In our study, the expression level of *BnaC06g29980D* was higher in No.73290 than in Zhongshuang11, which might promote flower formation. *BLH9,* a homeodomain transcription factor, contributes to spatial expression patterns of boundary genes and floral induction by *FT* [44]. Loss function of this gene impairs stem cell maintenance and blocks internode elongation and flowering [45]. *AT1G60340* belongs to the NAC family, whose proteins have a consensus sequence known as the NAC domain (*petunia NAM* and *Arabidopsis ATAF1*, *ATAF2*, and *CUC2*) [46]. Previous studies showed that mutated *NAM* (NO APICAL MERISTEM) genes failed to form SAM in *petunia* and that mutated *CUC2* causes defect in the formation of the SAM in *Arabidopsis* [46].

Phytohormones, such as auxin, cytokinins and gibberellin etc., play an important role in the shoot apical and floral meristem functions [35]. Of the above-mentioned nine candidates, *BnaA03g25890D* is homologous to *Arabidopsis ABP1,* which functions as an auxin receptor and its knockdown leads to an enhanced degradation of AUX/IAA repressors [47]. In current study, the expression level of *BnaA03g25890D* was lower in No.73290 than in Zhongshuang11, which might promote flower primordium initiation.

*BnaA03g29180D* is homologous to *ATSK12*, which encodes SHAGGY-like protein kinase involved in meristem organization [48]. *BnaA05g12220D* is homologous to *ASL5*, which encodes the protein containing a conserved LOB domain. The T-DNA insertion mutant of *ASL5* displayed lost apical dominance, abnormal inflorescence compare to wide type [49, 50]. In our study, the expression level of *BnaA03g29180D* and *BnaA05g12220D* in No.73290 was higher more than in Zhonghsuang11, which suggested that *BnaA03g29180D* and *BnaA05g12220D* may positively regulate the SAM development.

*BnaC02g02900D* is homologous to *TFL1* (encoding a small transcription cofactors), which controls inflorescence meristem identity and is involved in the floral initiation process [31, 51]. To date, the function of *TFL1* has been extensively studied in soybean [52], rose [53] and black cherry [54] etc., which all indicated its role in the transcription repression in floral development. In our study, the expression level of *BnaC02g02900D* was lower in No.73290 than in Zhongshuang11, which might promote floral development. *BnaA03g29810D* is homologous to *NSN1*, which encodes a nucleolar GTP-binding protein and is required for maintenance of inflorescence meristem identity and floral organ development.[55]. Loss function mutant of *NSN1* showed a smaller inflorescence meristem dome in comparison to the wide type plants at young stages [56]. In current study, *BnaA03g29810D* showed higher expression level in No.73290 than in Zhongshuang11, which might promote floral organ development. *BnaC06g28880D* is homologous to *TEL2*, which is similar to the terminal ear1 (*TE1*) gene in maize. The *te1* mutant displayed earlike appearance of the terminal inflorescence, which indicated that *TEL2* may contribute to normal inflorescence and lead to floral development [57]. The expression level of *BnaC06g28880D* was higher in No.73290 than in Zhongshuang11, which might promote inflorescence and flower development.

To further validate the nine candidate genes, their over-expression and knockout vector have been constructed and transformed in our laboratory.

## Conclusions

To investigate the genetic and molecular mechanism of pod number variation in rapeseed, we conducted QTL mapping and RNA sequencing, respectively, using the BnaZNRIL/BnaZNF_2_ populations and its two parents Zhongshuang11 and No.73290. As a result, 15 consensus-QTLs were identified through meta-analysis. Go and KEGG analysis of 9135 DEGs between the SAM of Zhongshuang11 and No.73290 provided new and further insights into pod number variation. An integrated analysis of QTLs and DEGs identified several promising candidates for these QTLs, which laid a solid foundation for further functional and mechanism research.

## Methods

### Population construction, field experiments and trait investigation

Two linkage populations, BnaZNF_2_ and BnaZNRIL, were used for mapping and integration of QTLs for pod number in our study. Both populations were derived from the two sequenced rapeseed cultivars, Zhongshuang11 (de novo sequencing) and No.73290 (re-sequencing) that showed significant difference on pod number. The BnaZNF_2_ population included 184 F_2_ individuals, which had been reported previously [4]. The BnaZNRIL population included 184 F_7_ generation RILs (recombinant inbred lines) derived from the above-mentioned 184 F_2_ individuals using single-seed descent.

The BnaZNRIL population was planted in Wuhan (Hu Bei province, China) and Zhengzhou (He Nan province, China) from Oct. 2012 to May 2013 and from Oct. 2013 to May 2014 (code W13RIL, Z13RIL, W14RIL, and Z13RIL, respectively) followed a randomized complete block design with two replications. Each block contained three rows, with 33 cm distance between rows and 16.7 cm distance between individuals. The field management followed standard agriculture practice. At maturity, 10 representative plants from the middle of the second row of each block were harvested.

Effective pod number was investigated according to the previous study [3]. Pod number included three categories: pods from the main inflorescence (PNm), branch inflorescence (PNb) and whole plant (PNw), respectively.

### SNP genotyping and linkage map construction

To genotype the BnaZNRIL population, The Brassica 60 K Illumina® Infinium SNP array was recently developed by the international *Brassica* Illumina SNP consortium. The array hybridization and data processing were carried out in Emei Tongde Co. (Beijing) according to the manufacturer’s recommendations. Leaf tissue from seedlings of BnaZNRIL population was used to extract genomic DNA according to the CTAB method [58].

The genetic linkage map was constructed using the software JoinMap 4.1 (https://www.kyazma.nl/index.php/JoinMap/) with a threshold for goodness-of-fit ≤5, recombination frequency of < 0.4 and minimum logarithm of odds (LOD) score of 2.0. The genetic distances were measured based on the Kosambi function. To avoid the potential errors, double-crossover events were checked.

### QTL mapping and meta-analysis

QTL mapping was performed using the composite interval mapping (CIM) method incorporated into WinQTLCart v2.5 software (http://statgen.ncsu.edu/qtlcart/WQTLCart.htm). The parameters including walk speed, number of control markers, window size and regression method were set to 1 cM, 5, 10 cM, and forward regression, respectively. Permutation analysis [4] with 1000 repetitions was used to determine the LOD threshold. The LOD value corresponding to *P* = 0.05 (3.0–4.6) was used to detected significant QTLs. Moreover, a lower LOD value corresponding to *P* = 0.10 (2.6–4.1) was employed to identify suggestive QTLs with small effects.

Meta-analysis was used to estimate the number and position of the true QTLs underlying the QTLs of the same or related traits, which were repeatedly detected in the different environments and/or populations [59]. QTLs repeatedly detected in the same population from different environments (year-location combinations) were first integrated into identified QTLs, and then QTLs repeatedly detected in the different populations were integrated into consensus-QTLs. Each identified and consensus-QTLs were named according to a previous study [60].

### SAM sampling and RNA isolation

To gain credible data, the SAMs were sampled from 10 representative individuals growing at the formation stage of floral bud primordia and then equally mixed for Zhongshuang11 and No.73290, respectively. Total RNA was isolated using an RNeasy Plant Mini Kit (Cat. 74124, Qiangen, Mississauga, ON) according to the manufacturer’s protocol. Then trace amount of DNA was treated with DNase I (Cat. 18068–015, Invitrogen, USA). In addition, the RNA quantity and quality were detected using a NanoDrop 1000 spectrophotometer (Thermo Fisher Scientific, USA), and the integrity of RNA was detected by electrophoresis with a 1% agarose gel in 1 ╳ TBE buffer at 70 V for 45 min.

### cDNA libraries construction and Illumina sequencing

Sequencing libraries were prepared using NEB Next Ultra*™* directional RNA Library Prep Kit for Illumina (San Diego, CA, USA) according to the manufacturer’s recommendations. Briefly, purification of mRNA was achieved from total RNA by using ploy-T oligo-attached magnetic beads. Then, adding the Illumina proprietary fragmentation buffer to the RNA samples, fragmentation of mRNA was completed under elevated temperature. Taking those fragments as templates, the first-strand cDNA was synthesized via using random hexamers and SuperScript II. The second-strand cDNA was synthesized by adding buffer, dNTPs, DNA polymerase I and RNase H to the reaction system. Subsequently, purification of the double-strand cDNA was performed using AMPure XP beads. The remaining overhangs were repaired via exonuclease/polymerase. Adenylation of the 3’ ends of cDNA fragments were conducted. Illumina PE adapter oligonucleotides were ligated to prepare for hybridization. 200-bp cDNA fragments were extracted using an AMPure XP system (Beckman Coulter, Beverly, CA, USA). DNA fragments were amplified by 12 cycles of PCR. After enrichment of DNA, the libraries were sequenced using Illumina Hiseq 2000 platform and raw data was generated. Adaptor, duplication and low quality sequences were removed to get clean data.

### Differential gene expression analysis

First, all reads of each library were mapped to the reference genome using the SOAP program [21] with the default parameters. Second, the uniquely mapped reads were selected for quantifying the abundance. Third, the expression level was normalized using the values of RPKM (reads per kilobase per million reads). According to Bioconductor [61] package, DEseq (http://www.bioconductor.org/packages/release/bioc/html/DESeq.html) was used to measure gene differential expression between Zhongshuang11 and No.73290. The absolute value of log2 (Ratio) ≥ 1 (under the criterion of *P* ≤ 0.01 and false discovery rate (FDR) ≤ 0.001) was used as threshold to assess the significance of gene expression difference.

### Functional annotation and category

Total DEGs were annotated based on BLSAT search in six public databases, including the protein family (Pfam) database (http://pfam.xfam.org/), InterPro database (http://www.ebi.ac.uk/interpro/entry/IPR001461), Swiss-Prot protein database (http://web.expasy.org/docs/swiss-prot_guideline.html), TrEMBLE database (http://web.expasy.org/docs/swiss-prot_guideline.html), the gene ontology (GO) database (http://geneontology.org/) and the Kyoto Encyclopedia of Genes and Genomes pathway (KEGG) database (http://www.genome.jp/kegg/pathway.html).

DEGs were functionally annotated using BLASTN with an E value cutoff of 1.0E-5 in TAIR 10 database. Then, The GO classification was performed using Classification SuperViewer Tool (http://bar.utoronto.ca/ntools/cgi-bin/ntools_classification_superviewer.cgi).

Pathway analysis was performed by searching against the Kyoto Encyclopedia of Gene and Genome (KEGG) pathway database with E value threshold of 1.0E-5.

### Validation of RNA sequencing data by qRT-PCR

Twelve genes were selected randomly to validate RNA sequencing data by quantitative real-time PCR (qRT-PCR). The primer pairs were designed using Primer 5.0. Details of primer pairs were listed in Additional file 7: Table S7. Total RNA extracted for transcriptome sequencing was used for conducting qRT-PCR. qRT-PCR was performed using SYBR® Select Master Mix (2X) according to manufacturers’ recommendation. UBC21 gene was used as an internal control to normalize transcript levels [62]. Real-time assay for each gene was performed with three independent biological replicates under identical conditions. Gene expression was calculated according to the previous study [63].

## Additional files

Additional file 1: Table S1. ANOVA analysis of pod number in four experiments. PNm/PNb/PNw was the abbreviation of pod number from the main inflorescence, branch inflorescence and whole plant (XLS 28 kb)
Additional file 2: Table S2. A. List of all detected QTLs at both significant and suggestive level for pod number. B. List of identified QTLs after deleting non-reproducible suggestive QTLs for pod number. C. Details of identified QTLs for pod number in the BnaZNRIL population in rapeseed. (XLSX 21 kb)
Additional file 3: Table S3. List of QTLs identified in the our and other studies for pod number in rapeseed. (XLSX 21 kb)
Additional file 4: Table S4. The differentially expressed genes (DEGs) between Zhongshuang11 and No.73290 samples using false discovery rate (FDR) < 0.05 and fold change > 2 as the significant cutoff. RPKM: reads per kilobase per million reads. (XLSX 1029 kb)
Additional file 5: Table S5. Annotation for DEGs (E value < 1E-5). The most matched one was used to annotate the rapeseed genes. (XLSX 218 kb)
Additional file 6: Table S6. 190 KEGG pathways represented by DEGs between Zhongshuang11 and No.73290. (XLSX 128 kb)
Additional file 7: Table S7. Gene-specific primers for quantitative real-time PCR. UBC21 is the internal reference gene. F and R denote forward and reverse primers. (XLSX 10 kb)

## Abbreviations

DEG: Differentially expressed gene
FDR: False discovery rate
FM: Floral meristem
GO: Gene ontology
IM: Inflorescence meristem
KEGG: Kyoto encyclopedia of genes and genomes
QTL: Quantitative trait loci
RIL: Recombinant inbred line
RPKM: Reads per kilobase per million reads
SAM: Shoot apical meristem
SNP: Single nucleotide polymorphism

## References

[1] Diepenbrock W (2000). Yield analysis of winter oilseed rape (Brassica napus L.): a review. Field Crop Res.

[2] Li Y, Shen J, Wang T, Chen Q, Zhang X, Fu T, Meng J, Tu J, Ma C (2007). QTL analysis of yield-related traits and their association with functional markers in Brassica napus L. Aust J Agric Res.

[3] Shi J, Li R, Qiu D, Jiang C, Long Y, Morgan C, Bancroft I, Zhao J, Meng J (2009). Unraveling the complex trait of crop yield with quantitative trait loci mapping in Brassica napus. Genetics.

[4] Shi J, Zhan J, Yang Y, Ye J, Huang S, Li R, Wang X, Liu G, Wang H (2015). Linkage and regional association analysis reveal two new tightly-linked major-QTLs for pod number and seed number per pod in rapeseed (Brassica napus L.). Sci Rep.

[5] Qu B, Zhang W, Chen XH, Li N, Cui N, Li TL (2010). Research progress of flower bud differentiation mechanism of plant. Chin Agric Sci Bull.

[6] Vaddepalli P, Scholz S, Schneitz K (2015). Pattern formation during early floral development. Curr Opin Genet Develop.

[7] Dodsworth S (2009). A diverse and intricate signalling network regulates stem cell fate in the shoot apical meristem. Dev Biol.

[8] Gaillochet C, Daum G, Lohmann JU (2015). O cell, where art thou? The mechanisms of shoot meristem patterning. Curr Opin Plant Biol.

[9] Holt AL, van Haperen JM, Groot EP, Laux T (2014). Signaling in shoot and flower meristems of Arabidopsis thaliana. Curr Opin Plant Biol.

[10] Zadnikova P, Simon R (2014). How boundaries control plant development. Curr Opin Plant Biol.

[11] Yruela I (2015). Plant development regulation: Overview and perspectives. J Plant Physiol.

[12] Suzaki T, Sato M, Ashikari M, Miyoshi M, Nagato Y, Hirano HY (2004). The gene FLORAL ORGAN NUMBER1 regulates floral meristem size in rice and encodes a leucine-rich repeat receptor kinase orthologous to Arabidopsis CLAVATA1. Development.

[13] Suzaki T, Toriba T, Fujimoto M, Tsutsumi N, Kitano H, Hirano HY (2006). Conservation and diversification of meristem maintenance mechanism in Oryza sativa: Function of the FLORAL ORGAN NUMBER2 gene. Plant Cell Physiol.

[14] Jiang L, Zhang W, Xia Z, Jiang G, Qian Q, Li A, Cheng Z, Zhu L, Mao L, Zhai W (2007). A paracentric inversion suppresses genetic recombination at the FON3 locus with breakpoints corresponding to sequence gaps on rice chromosome 11L. Mol Genet Genomics.

[15] Chu H, Qian Q, Liang W, Yin C, Tan H, Yao X, Yuan Z, Yang J, Huang H, Luo D (2006). The floral organ number4 gene encoding a putative ortholog of Arabidopsis CLAVATA3 regulates apical meristem size in rice. Plant Physiol.

[16] Xu J, Song X, Cheng Y, Zou X, Zeng L, Qiao X, Lu G, Fu G, Qu Z, Zhang X (2014). Identification of QTLs for branch number in oilseed rape (Brassica napus L.). J Genet Genomics.

[17] Ozsolak F, Milos PM (2011). RNA sequencing: advances, challenges and opportunities. Nat Rev Genet.

[18] Manuel G, Grabherr MG, Mitchell G, Cole T (2011). Computational methods for transcriptome annotation and quantification using RNA-seq. Nat Methods.

[19] Xu HM, Kong XD, Chen F, Huang JX, Lou XY, Zhao JY (2015). Transcriptome analysis of Brassica napus pod using RNA-Seq and identification of lipid-related candidate genes. BMC Genomics.

[20] Chalhoub B, Denoeud F, Liu S, Parkin IA, Tang H, Wang X, Chiquet J, Belcram H, Tong C, Samans B (2014). Plant genetics. Early allopolyploid evolution in the post-Neolithic Brassica napus oilseed genome. Science.

[21] Li R, Li Y, Kristiansen K, Wang J (2008). SOAP: short oligonucleotide alignment program. Bioinformatics.

[22] Wagner GP, Kin K, Lynch VJ (2012). Measurement of mRNA abundance using RNA-seq data: RPKM measure is inconsistent among samples. Theory Biosci.

[23] Jiang Y, Deyholos MK (2010). Transcriptome analysis of secondary-wall-enriched seed coat tissues of canola (Brassica napus L.). Plant Cell Rep.

[24] Herrmann J, Ravilious GE, McKinney SE, Westfall CS, Lee SG, Baraniecka P, Giovannetti M, Kopriva S, Krishnan HB, Jez JM (2014). Structure and mechanism of soybean ATP sulfurylase and the committed step in plant sulfur assimilation. J Biol Chem.

[25] Hao JH, Qi HY, Yan N, Wang HX (2008). Advances in researches on flower bud differentiation of horticultural crops. Agric Sci Technol Equip.

[26] Galston AW (1983). Polyamines as Modulators of Plant Development. Bioscience.

[27] Sun WQ, Chu MY (1990). Study on content variations of endogenous amino acids at the critical period of floral initiation in MEI-FLOWER. Acta Agriculiurae Shanghai.

[28] Sassi M, Ali O, Boudon F, Cloarec G, Abad U, Cellier C, Chen X, Gilles B, Milani P, Friml J (2014). An auxin-mediated shift toward growth isotropy promotes organ formation at the shoot meristem in Arabidopsis. Curr Biol.

[29] Wagner D, Sablowski RW, Meyerowitz EM (1999). Transcriptional activation of APETALA1 by LEAFY. Science.

[30] Weigel D, Alvarez J, Smyth DR, Yanofsky MF, Meyerowitz EM (1992). LEAFY controls floral meristem identity in Arabidopsis. Cell.

[31] Baumann K, Venail J, Berbel A, Domenech MJ, Money T, Conti L, Hanzawa Y, Madueno F, Bradley D (2015). Changing the spatial pattern of TFL1 expression reveals its key role in the shoot meristem in controlling Arabidopsis flowering architecture. J Exp Bot.

[32] Takacs EM, Li J, Du C, Ponnala L, Janick-Buckner D, Yu J, Muehlbauer GJ, Schnable PS, Timmermans MCP, Sun Q (2012). Ontogeny of the Maize Shoot Apical Meristem. Plant Cell.

[33] Wong CE, Singh MB, Bhalla PL (2013). The dynamics of soybean leaf and shoot apical meristem transcriptome undergoing floral initiation process. PLoS One.

[34] Murray JA, Jones A, Godin C, Traas J (2012). Systems analysis of shoot apical meristem growth and development: integrating hormonal and mechanical signaling. Plant Cell.

[35] Shani E, Yanai O, Ori N (2006). The role of hormones in shoot apical meristem function. Curr Opin Plant Biol.

[36] Gendron JM, Jiang-Shu L, Min F, Ming-Yi B, Stephan W, Springer PS, Kathryn B, Zhi-Yong W (2012). Brassinosteroids regulate organ boundary formation in the shoot apical meristem of Arabidopsis. Proc Natl Acad Sci U S A.

[37] Han Y, Gao S, Muegge K, Zhang W, Zhou B (2015). Advanced Applications of RNA Sequencing and Challenges. Bioinformatics Biol Insights.

[38] Wang Z, Gerstein MM (2009). RNA-Seq: a revolutionary tool for transcriptomics. Nat Rev Genet.

[39] Fernandez-Lozano A, Yuste-Lisbona FJ, Perez-Martin F, Pineda B, Moreno V, Lozano R, Angosto T (2015). Mutation at the tomato excessive number of floral organs (ENO) locus impairs floral meristem development, thus promoting an increased number of floral organs and fruit size. Plant Sci.

[40] Sun B, Ito T (2015). Regulation of floral stem cell termination in Arabidopsis. Front Plant Sci.

[41] Han Y, Jiao Y. APETALA1 establishes determinate floral meristem through regulating cytokinins homeostasis in Arabidopsis. Plant Signal Beh. 2015;10(11):e989039. 10.4161/15592324.2014.989039 PMC4883926 26359644

[42] Bowman JL, Alvarez J, Weigel D, Meyerowitz EM, Smyth DR (1991). Control of flower development in Arabidopsis thaliana by APETALA1 and interacting genes. Development.

[43] Irish VF, Sussex IM (1990). Function of the apetala-1 gene during Arabidopsis floral development. Plant Cell.

[44] Andres F, Romera-Branchat M, Martinez-Gallegos R, Patel V, Schneeberger K, Jang S, Altmuller J, Nurnberg P, Coupland G (2015). Floral Induction in Arabidopsis by FLOWERING LOCUS T Requires Direct Repression of BLADE-ON-PETIOLE Genes by the Homeodomain Protein PENNYWISE. Plant Physiol.

[45] Khan M, Ragni L, Tabb P, Salasini BC, Chatfield S, Datla R, Lock J, Kuai X, Despres C, Proveniers M (2015). Repression of Lateral Organ Boundary Genes by PENNYWISE and POUND-FOOLISH Is Essential for Meristem Maintenance and Flowering in Arabidopsis. Plant Physiol.

[46] Ooka H, Satoh K, Doi K, Nagata T, Otomo Y, Murakami K, Matsubara K, Osato N, Kawai J, Carninci P (2003). Comprehensive analysis of NAC family genes in Oryza sativa and Arabidopsis thaliana. DNA Res.

[47] Tromas A, Paque S, Stierle V, Quettier AL, Muller P, Lechner E, Genschik P, Perrot-Rechenmann C (2013). Auxin-binding protein 1 is a negative regulator of the SCF(TIR1/AFB) pathway. Nat Commun.

[48] Dornelas MC, Van Lammeren AA, Kreis M (2000). Arabidopsis thaliana SHAGGY-related protein kinases (AtSK11 and 12) function in perianth and gynoecium development. Plant J.

[49] Shuai B, Reynaga-Pena CG, Springer PS (2002). The lateral organ boundaries gene defines a novel, plant-specific gene family. Plant Physiol.

[50] Nakazawa M, Ichikawa T, Ishikawa A, Kobayashi H, Tsuhara Y, Kawashima M, Suzuki K, Muto S, Matsui M (2003). Activation tagging, a novel tool to dissect the functions of a gene family. Plant J.

[51] Ho WW, Weigel D (2014). Structural features determining flower-promoting activity of Arabidopsis FLOWERING LOCUS T. Plant Cell.

[52] Ping J, Liu Y, Sun L, Zhao M, Li Y, She M, Sui Y, Lin F, Liu X, Tang Z (2014). Dt2Is a Gain-of-Function MADS-Domain Factor Gene That Specifies Semideterminacy in Soybean. Plant Cell.

[53] Randoux M, Daviere JM, Jeauffre J, Thouroude T, Pierre S, Toualbia Y, Perrotte J, Reynoird JP, Jammes MJ, Hibrand-Saint Oyant L (2014). RoKSN, a floral repressor, forms protein complexes with RoFD and RoFT to regulate vegetative and reproductive development in rose. New Phytol.

[54] Wang Y, Pijut PM (2013). Isolation and characterization of a TERMINAL FLOWER 1 homolog from Prunus serotina Ehrh. Tree Physiol.

[55] Wang X, Xie B, Zhu M, Zhang Z, Hong Z (2011). Nucleostemin-like 1 is required for embryogenesis and leaf development in Arabidopsis. Plant Mol Biol.

[56] Wang X, Gingrich DK, Deng Y, Hong Z (2012). A nucleostemin-like GTPase required for normal apical and floral meristem development in Arabidopsis. Mol Biol Cell.

[57] Veit B, Briggs SP, Schmidt RJ, Yanofsky MF, Hake S (1998). Regulation of leaf initiation by the terminal ear 1 gene of maize. Nature.

[58] Doyle JJ (1987). A rapid DNA isolation procedure for small quantities of fresh leaf tissue. Phytochem Bull.

[59] Goffinet B, Gerber S (2000). Quantitative trait loci: a meta-analysis. Genetics.

[60] Li N, Shi J, Wang X, Liu G, Wang H (2014). A combined linkage and regional association mapping validation and fine mapping of two major pleiotropic QTLs for seed weight and silique length in rapeseed (Brassica napus L.). BMC Plant Biol.

[61] Gentleman RC, Carey VJ, Bates DM, Bolstad B, Dettling M, Dudoit S, Ellis B, Gautier L, Ge Y, Gentry J (2004). Bioconductor: open software development for computational biology and bioinformatics. Genome Biol.

[62] Chen X, Truksa M, Shah S, Weselake RJ (2010). A survey of quantitative real-time polymerase chain reaction internal reference genes for expression studies in Brassica napus. Anal Biochem.

[63] Livak KJ, Schmittgen TD (2001). Analysis of relative gene expression data using real-time quantitative PCR and the 2(−Delta Delta C(T)) Method. Methods.

